# Prediction of Mild Cognitive Impairment Status: Pilot Study of Machine Learning Models Based on Longitudinal Data From Fitness Trackers

**DOI:** 10.2196/55575

**Published:** 2024-07-18

**Authors:** Qidi Xu, Yejin Kim, Karen Chung, Paul Schulz, Assaf Gottlieb

**Affiliations:** 1 McWilliams School of Biomedical Informatics University of Texas Health Science Center at Houston Houston, TX United States; 2 McGovern Medical School University of Texas Health Science Center at Houston Houston, TX United States

**Keywords:** mild cognitive impairment, Fitbits, fitness trackers, sleep, physical activity

## Abstract

**Background:**

Early signs of Alzheimer disease (AD) are difficult to detect, causing diagnoses to be significantly delayed to time points when brain damage has already occurred and current experimental treatments have little effect on slowing disease progression. Tracking cognitive decline at early stages is critical for patients to make lifestyle changes and consider new and experimental therapies. Frequently studied biomarkers are invasive and costly and are limited for predicting conversion from normal to mild cognitive impairment (MCI).

**Objective:**

This study aimed to use data collected from fitness trackers to predict MCI status.

**Methods:**

In this pilot study, fitness trackers were worn by 20 participants: 12 patients with MCI and 8 age-matched controls. We collected physical activity, heart rate, and sleep data from each participant for up to 1 month and further developed a machine learning model to predict MCI status.

**Results:**

Our machine learning model was able to perfectly separate between MCI and controls (area under the curve=1.0). The top predictive features from the model included peak, cardio, and fat burn heart rate zones; resting heart rate; average deep sleep time; and total light activity time.

**Conclusions:**

Our results suggest that a longitudinal digital biomarker differentiates between controls and patients with MCI in a very cost-effective and noninvasive way and hence may be very useful for identifying patients with very early AD who can benefit from clinical trials and new, disease-modifying therapies.

## Introduction

Alzheimer disease (AD) is the sixth leading cause of death in the United States and incurs a heavy economic burden of US $257 billion in direct costs [1]. The numbers are staggering—11.3% of Americans aged 65 years and older have Alzheimer dementia, and more than twice as many are anticipated to have Alzheimer dementia by 2050 [1]. Moreover, estimates suggest that 46.7 million Americans are already in a preclinical AD stage [2]. AD diagnoses are often significantly delayed to time points when brain damage has already occurred, owing to difficulty in detecting early signs of AD due to cost and effort, which makes it critical to identify efficient methods for early detection of AD signs. One pillar of AD programs, like the National Alzheimer's Project Act, focuses on early diagnosis of AD, as it allows patients to make lifestyle changes and consider new treatment options. Early diagnosis and identification of the trajectory of cognitive decline would also allow pharmaceutical companies to develop better therapeutics to delay or halt the progression to AD and support enrollment in experimental trials.

A growing body of evidence indicates that cognitive, sensory, and motor changes may precede clinical manifestations of AD by several years [3]. Validated neuropsychological or cognitive tests are often less effective in the earliest stages of the disease and are impractical for frequent testing [4,5]. Frequently studied biomarkers, such as detection of beta-amyloid and tau proteins in cerebrospinal fluid [6,7] or using structural magnetic resonance imaging (MRI) [8] or positron emission tomography (PET) molecular imaging [9] promise earlier detection of disease, but these tests are infrequently performed in clinical settings since they are invasive or costly. Additionally, not all individuals with amyloid plaques will go on to develop dementia [10]. This calls for inexpensive and noninvasive methods that can provide clinicians with ways to augment these biomarkers and help triage patients eligible for these expensive and invasive tests.

One biomarker that has been associated with early changes in patients with mild cognitive impairment (MCI) is disruption of sleep. Sleep disturbance is prevalent in mild cognitive decline and has been previously associated with cognitive decline in several meta-analyses when compared across different stages of cognitive status [11,12]. Previous studies suggest that poor sleep quality is associated with the presence of amyloid plaques in cognitively healthy individuals in AD-sensitive brain regions [13] and that even 1 night of sleep deprivation affects the levels of amyloid-β in the cerebrospinal fluid of healthy middle-aged men [14]. Other studies suggest that poor sleep may also be associated with increased levels of tau in the cerebrospinal fluid of cognitively normal people [15] and participants with the highest levels of tau had the lowest levels of nonrapid eye movement (NREM) across different cognitive statuses from unimpaired to maximal impairment [16]. Finally, high-frequency heart rate variability (HRV), especially during NREM sleep, may be an early biomarker for dementia detection when comparing older adults with MCI to patients with subjective cognitive impairment [17].

A growing body of evidence also strongly suggests that physical exercise may attenuate cognitive impairment and reduce the risk of AD via a variety of mechanisms [18] and that some preventable lifestyle-related risk factors such as a sedentary lifestyle and lack of physical activity increase the risk of dementia and AD [19,20]. Proposed mechanisms that may be responsible for this effect include increased production of neurotrophic factors and neurogenesis, increased cerebral blood flow, reduced risk of cardiovascular diseases [21], increased cortical thickness, and enhanced cognitive function through improved cardiorespiratory fitness in MCI patients [22]. Although physical activity does not attenuate cognitive impairment, low levels are a risk factor for dementia [23].

Although previous studies have shown that sleep and physical activity are individually associated with AD [11,12,17,20,24-26], their measurements were either based on collection via self-reported questionnaires, which tend to overestimate objective measurements [27-30], or collected over very short periods of time (typically 2-6 days) via an actigraph [15,16,25], limiting the ability to assess temporal variability and track gradual changes in these traits. Additionally, actigraphs typically do not measure heart rate, and acceleration features show only modest performance in differentiating between sleep stages [31,32]. Wearable technologies present a unique opportunity to capture these early changes preceding clinical decline while addressing limitations of the aforementioned biomarkers and circumvent inaccuracy in self-reported traits, but studies have thus far relied mostly on costly medical devices that were used only for short time periods. A review discussing the potential of digital markers for early detection of AD indicated that accurate algorithms require longitudinal observational studies with confirmed MCI and healthy controls [3].

Using cost-effective, widely available fitness trackers (Fitbit Charge 4), we captured sleep, physical activity, and heart rate. Passive collection is well-suited for older adults, especially those who are already diagnosed with MCI, as it avoids relying on subjective recollection and does not interfere with their day-to-day activities. Studies have determined that Fitbits provide similar estimates of sleep outside the laboratory to a research-grade actigraph [33], and another study showed that consumer-grade multimodal sensor streams can detect MCI status [34].

Here, we report a pilot study that demonstrates that sleep, physical activity, and heart rate recorded from a fitness tracker can distinguish between MCI and age-matched controls with a high accuracy. Furthermore, we show that 11 days of recording is sufficient to reach high accuracy. These methods may be used for early detection of decline in cognitive function, which can prompt clinicians to conduct further testing.

## Methods

### Recruitment

This study included participants who visited the University of Texas Health Science Center at Houston (UTHealth) Neurosciences Neurocognitive Disorders Center (NDC) at Houston, TX between January 2022 and September 2023. Per the approved recruitment protocol, patients and their spouses visiting the NDC who matched the inclusion criteria (see the Cohort Definition section) were approached by a study coordinator to participate in a fitness tracking study.

Interested participants were consented by the study coordinator in a private room and were given a written consent form and time to ask any questions. The study was discussed, including the purpose, procedures, associated risks and benefits, time commitments, and costs. Participants were also told that participation was voluntary and they could withdraw at any time. If the participant agreed to be in the study, the participant signed the informed consent form. A signed and dated copy was given to the participant. The same procedures were followed for consent of the study partner. Patient privacy was protected by assigning unique study IDs to each participant, and the mapping was available only to the study coordinator.

After participants consented, the recruiting study coordinator set up their fitness tracker (Fitbit Charge 4) and installed the corresponding free Fitbit app on the participant’s smartphone. Participants were also provided with instructions on how to wear and charge the fitness tracker and were instructed to keep their Bluetooth on their phones so the Fitbit app could synchronize with the fitness tracker. Each participant wore their fitness tracker between 2 consecutive visits to the clinic, returning it during their next visit.

### Ethical Considerations

The study was approved by the UTHealth institutional review board (HSC-MS-21-0719).

### Cohort Definition

#### Cohort

The recruited cohort included 12 cases, including 11 patients with amnestic MCI and 1 diagnosed with early AD. Additionally, 8 age-matched healthy controls were recruited. All the recruited controls were spouses of the recruited MCI, enabling minimization of variability in lifestyle affecting physical activity and sleep times. Having the spouse wear a fitness tracker concomitantly also helped verify that they were charged on time.

#### Inclusion Criteria

The eligibility criteria included cases and controls aged 60 years to 80 years.

#### MCI Diagnosis

Cases were diagnosed by a neurologist who runs the NDC clinic with 35 years of experience diagnosing MCI. The diagnostic process starts with surveying the patient’s previous medical history (eg, comorbidities), patient background, family history, social history, sleep patterns, mood issues, and other neurologic changes, such as gait, from the patient and their family. The clinical team asks directed questions to better identify areas of change in cognition, mood, behavior, and activities of daily living. Screening tests are administered, including the Montreal Cognitive Assessment [35] and Mini Mental State Examination [36] to identify the level of cognitive impairment. Depending on these results, other tests are ordered to clarify the diagnosis and rule out alternative diagnoses. The initial tests most often include neuropsychological testing [37], MRI of the brain [38], and blood work. They can, however, also include sleep studies, electroencephalograms (EEGs), and lumbar punctures. The neurologist meets with patients and families and reviews the results. If appropriate, other testing is then ordered, including fluorodeoxyglucose PET [39], amyloid PET [40], or spinal taps. The neurologist or staff then meet the patient to explain the test conclusions. When testing is consistent with MCI due to AD pathology, patients were considered for inclusion in this study.

#### Exclusion Criteria

Participants were excluded if they were hospitalized within 1 month prior to screening, had suspected or known drug or alcohol abuse (ie, more than approximately 60 g of alcohol [approximately 1 liter of beer or 0.5 liter of wine] per day), had acute intercurrent infections, or were unable or unwilling to comply with the protocol and follow-up requirements. Patients taking sleep-inducing medication such as benzodiazepines were also excluded.

A list of comorbidities and prescribed medications used by the patients with MCI is available in Tables S1 and S2 in Multimedia Appendix 1. We did not collect this information for the controls since the controls were not patients of the NDC.

### Data Collection

Data, including daily summaries of sleep, activity, and heart rate, were collected through the Fitbit application programming interface using proprietary scripts coded in Python 3.9.

For activities, we adopted the All of Us definition for a valid day as a participant wearing the Fitbit for at least 10 hours per day and reporting at least 100 steps per day [41]. Days not considered valid based on this threshold were excluded, including the days of the clinic visits when the participants were recruited or returned their fitness trackers.

Fitbit algorithms differentiate between the main sleep epoch and naps with regard to capturing sleep stages. For the main sleep epoch, Fitbit measures periods of light, deep, rapid eye movement (REM), and wake stages. For naps, Fitbit detects awake, asleep, and restless stages. Due to differences in captured features, the main sleep epoch and naps were separated for the purpose of modeling.

### Assessing the Sex Imbalance Effect on Model Performance

The sex distribution in the MCI and control groups was imbalanced, with 4 (4/12, 33%) women in the MCI group and 7 (7/8, 88%) women in the control group. To isolate the effect of sex on predictions, we modeled the MCI prediction as the following regression model:

*MCI status*=*a*·*sex*+*b*

After obtaining the coefficient *a* and residual *b*, a separate regression model was built on the residuals *b* as the dependent variable. This 2-step approach discerned whether other features, apart from sex, could effectively capture the unexplained variability in the residuals. The logistic regression model of sex yielded an area under the curve (AUC) value of 0.58, and a regression model based on the residuals *b* obtained an R^2^ of 0.96, which meant that sex plays a minor role in differentiating between cases and controls. This led us to exclude sex from future analysis to reduce bias from recruitment imbalance.

### Aggregation Model and Daily Model

We tested 2 sets of prediction schemes (Figure S1 in Multimedia Appendix 1). The first scheme (“aggregation model”) aggregated the daily records for each participant to a single record. Summary statistics, including minimal, maximal, mean, sum, standard deviation, and skewness were applied to the daily data. The final set of features included age and 329 engineered summary statistics features from the fitness trackers.

The second scheme (“daily model”) classified individual days per participant. The classifier predicted MCI status per day of each participant, followed by a majority vote on the percent of days predicted to be MCI per participant.

We included age in the aggregation model, although there was no statistically significant difference between MCI and controls. To avoid introducing artificial bias into the daily model resulting from the difference in the number of days measured per participant (ie, the number of days a specific participant age was replicated depends on the number of days the fitness tracker was worn), we excluded age from the daily model, but this exclusion did not alter the performance.

### Feature Selection

In order to deal with the large number of variables, we built a prediction model to select important features. These selected features were subsequently fed into a final prediction model.

We tested several machine learning algorithms to select the features based on the best performing algorithm, including Gaussian naïve Bayes, logistic regression, support vector machine (SVM), random forest, XGBoost, and CatBoost. In each aggregation and daily data set, we used all features to train classifiers using 5-fold cross-validation.

For each machine learning algorithm, we calculated Shapley Additive Explanations (SHAP) values to illuminate the impact of each feature on the model's prediction. We conducted independent feature selection on each training fold (alpha=0.02) and tested them on their respective testing folds. Finally, we identified features as important if they appeared in at least 2 of the 5 folds.

### Final Models and Evaluation

The final aggregation and daily models were trained using only the features selected in the feature selection stage. We built prediction models with naïve Bayes, logistic regression, SVM, random forest, XGBoost, and CatBoost.

In order to gain robustness, we conducted both 5 repeats of 5-fold cross-validation, each time with different data splits, and a leave-one-out cross-validation technique. The mean AUC values across the 5 repeats served as the standardized metric to identify the best model. When dealing with daily models, we used the majority vote on the number of days a participant had days classified as MCI, classifying a participant as MCI if the proportion of days classified as MCI out of their total number of measured days exceeded 50%. The proportion of MCI-classified days per participant also served for computing the AUC value for each testing fold. As before, we computed the mean AUC values to select the most effective model.

### Statistical Analysis

Age differences between the MCI patients and controls were computed using the Wilcoxon ranked sum test, and sex differences were calculated using the Fisher exact test.

## Results

### Participant Characteristics

**Figure 1:**
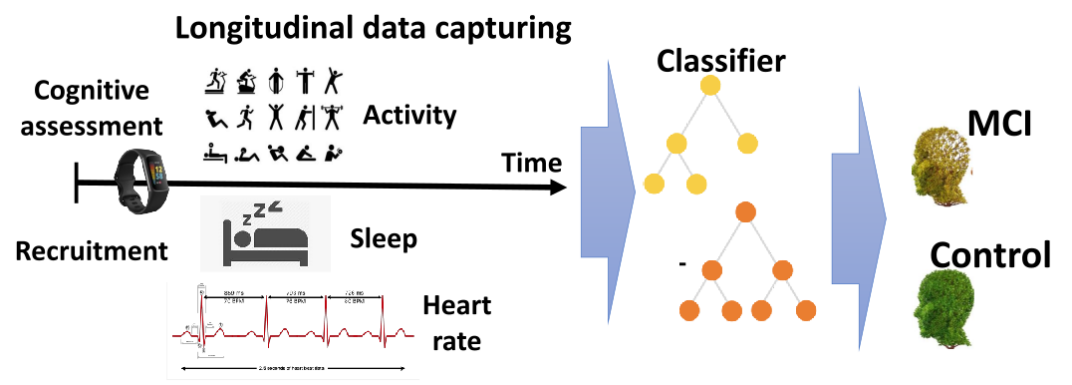
Illustration of data collection over a period of ~1 month, followed by a machine learning classifier to detect mild cognitive impairment (MCI).

We collected fitness tracker data from 12 patients with MCI or early AD and 8 age-matched controls between 2 clinic visits (as described in the Methods section; Figure 1).

After removing partial activity days (for more information, see the Methods section), a total of 530 tracked heart rate days (MCI: mean 25.6, SD 10.1 days; control: mean 27.9, SD 8.5 days), 510 activity days (MCI: mean 24.4, SD 10 days; control: mean 27.1, SD 8.2 days), and 499 sleep days or nights (MCI: mean 23.6, SD 8.8 days; control: mean 27, SD 9 days or nights; Figure S2 in Multimedia Appendix 1) remained. One MCI patient returned their fitness tracker early (Figure S2 in Multimedia Appendix 1).

The average age of the patients in the MCI group was higher by 1.3 years than the control group’s age (Table 1), but the difference was not statistically significant (*P*=.6). Additionally, 55% (11/20) of all the participants were women. However, significantly more women were recruited to the control group than to the MCI group (*P=*.03; Table 1).

**Table 1:** Demographic characteristics of the recruited patients to wear the fitness tracker (all participants were White).

Group	Participants, n	Women, n	Age (years), mean (SD)
Mild cognitive impairment	12	4	71.8 (7.3)
Controls	8	7	70.5 (5.7)

### Predicting MCI Status

We tested models for predicting MCI status that considered 2 types of processing the data: a model that aggregated the daily data per participant and a model that classified individual daily records (see the Methods section). We also tested the effect of using different combinations of data modalities from the 3 groups of measures—sleep, physical activity, and heart rate.

In the aggregation model, we chose to use CatBoost as the preliminary model because it outperformed other algorithms on our aggregation data, while SVM was chosen in the daily model due to its superior performance in daily data.

Regarding the aggregation model, different machine learning algorithms obtained the top accuracy for some data modality combinations of activity, heart rate, and sleep data (Tables S3 and S4 in Multimedia Appendix 1). However, the CatBoost algorithm was the most consistent algorithm at achieving high accuracy across combinations of modalities and across different cross-validation schemes.

**Figure 2:**
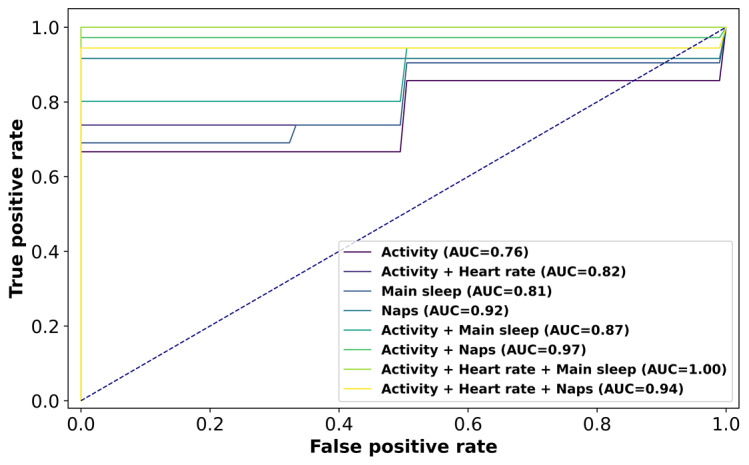
Receiver operating characteristic curve for 5-fold cross-validation of the CatBoost classifier; different curves correspond to different combinations of data modalities captured by fitness trackers. AUC: area under the curve.

The classification performance of different combinations of data modalities is displayed in Table 2. Adding data modalities increases the accuracy of the algorithm, and the combination of physical activity, heart rate, and main sleep achieved perfect separation (AUC=1.0; Table 2; Figure 2; Figure S3 in Multimedia Appendix 1). The combination of physical activity, heart rate, and naps also achieved good separation between MCI and controls (AUC=0.94; Table 2); however, it included only 15 participants (15/20, 75%: 11 MCI and 4 controls) that had taken any nap during the time they wore the fitness tracker.

**Table 2:** Performance of classifiers differentiating between patients with mild cognitive impairment and controls using different combinations of data modalities captured by fitness trackers for an aggregated data model.

Data	Important features selected, n	AUC^a^ of the repeated 5-fold CV^b^, mean	AUC of the LOOCV^c^
Activities	15	0.76	0.58
Activities + heart rate	10	0.82	0.82
Main sleep	15	0.81	0.77
Naps^d^	6	0.91	0.86
Activities + main sleep	15	0.87	0.84
Activities + naps^d^	11	0.97	0.93
Activities + heart rate + main sleep	15	1.0	1.0
Activities + heart rate + naps^d^	13	0.94	0.88

^a^AUC: area under the curve.

^b^CV: cross-validation.

^c^LOOCV: leave-one-out cross-validation.

^d^Classification of 15 participants (11 MCI and 4 controls) who had recorded naps within the time window.

**Figure 3:**
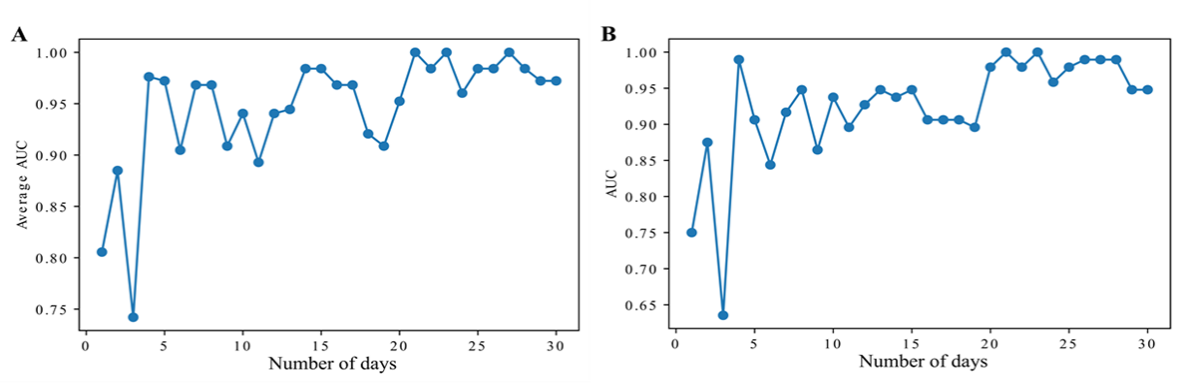
The machine learning model performance (area under the curve [AUC]) as a function of the maximal number of days recorded from fitness trackers for each participant in (A) 5-fold cross-validation and (B) leave one out cross-validation.

As a corollary question, we were interested in knowing the minimal number of days that a fitness tracker needed to be worn in order to obtain sufficient performance from the models. Using the same models described before, we observed that, after only 11 days of wearing it, the classification performance surpassed an AUC of 0.90 (Figure 3).

Regarding the daily model, we evaluated the accuracy of classifiers when classifying each day and taking the majority vote per participant (see the Methods section). The daily models performed worse than the aggregation models (Table S5 in Multimedia Appendix 1). Specifically for naps, many participants who had any naps had fewer than 3 days on which these naps occurred, contributing to much lower performance in daily combination compared with the aggregation data sets.

### Features Predictive of MCI Status

The selected features predictive of MCI status differed between different data modalities.

#### Aggregated Model

**Figure 4:**
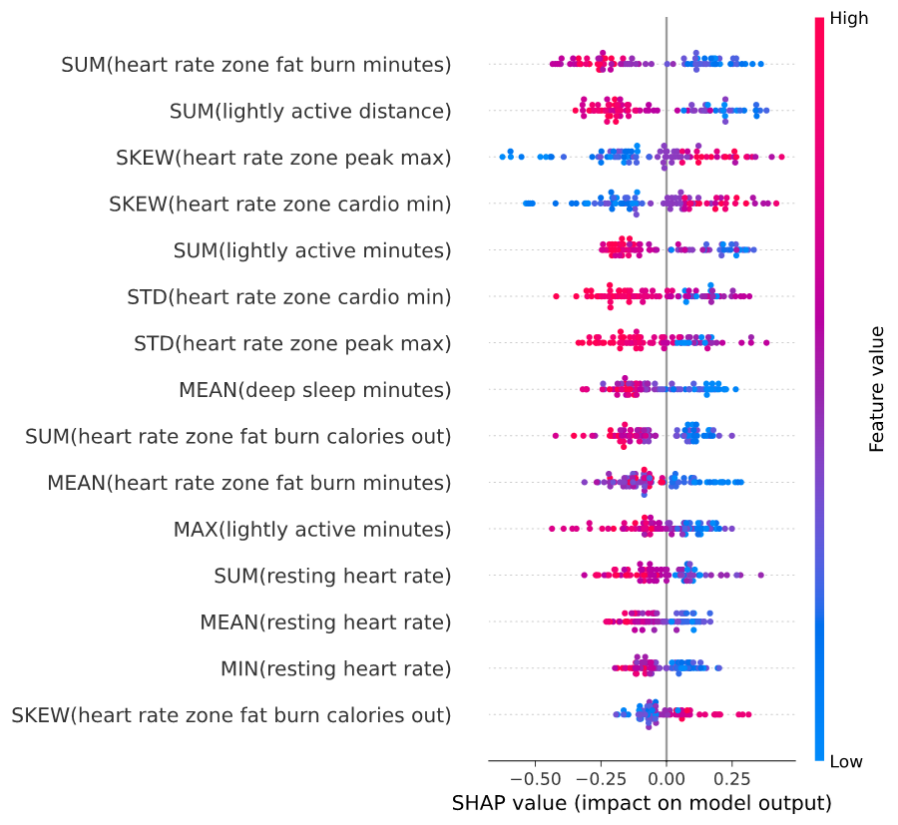
Top features captured from fitness trackers and used in the model to differentiate between mild cognitive impairment cases and healthy controls using aggregated daily data. SHAP: Shapley Additive Explanations.

The aggregated model included 15 selected features related to sleep (deep sleep minutes), heart rate (resting heart rate and cardio, fat burn, and peak heart rate zones), and physical activity (light active minutes and distance; Figure 4; Table S6 in Multimedia Appendix 1). When considering naps instead of main sleep (including only 15 participants who had naps; see the Methods section), we found 13 top features, including more features from physical activity and fewer features from heart rate (Table S6 in Multimedia Appendix 1). Notably, most of the top features from heart rate and physical activity data types were different when we replaced main sleep with naps, but lightly active physical activity minutes and distance and heart rate zone cardio time were common to the set of top features from the 2 data modalities.

#### Daily Model

The top performing daily data features included 13 top-selected features from sleep (eg, average of light sleep and REM minutes), heart rate (resting heart rate and several heart rate zones), and physical activity (calories expended and lightly active minutes; Table S7 in Multimedia Appendix 1). In contrast to the aggregated model, the models using main sleep and naps shared multiple features, including physical activity light and very active activities; calories out; and several heart rate zones, including resting heart rate and cardio, fat burn, and out-of-range zones.

## Discussion

### Principal Findings

In this pilot study, we recruited and tracked 20 MCI and healthy control participants using fitness trackers for a typical time of 2 weeks to 4 weeks in order to test the ability of the data captured by the fitness tracker to predict MCI status. We tested the use of features from physical activity, sleep, and heart rate measurements and trained a machine learning classifier to predict MCI status, obtaining perfect separation in predicting MCI status (AUC=1.0) when all the data types were combined. We compared 2 models with different engineered features: a model that classifies MCI status based on aggregated daily data for each participant and a model that classified individual days and determined MCI status based on a majority of days classified as MCI or control. We found that the model aggregating all days into a set of variables per participant performed better than when classifying each day in a daily model.

The top features identified in the aggregated model agree with those in previously published studies. The majority of the top features were related to heart rate zones, including peak, cardio, and fat burn zones. A systematic review of HRV and cognitive function suggested that HRV is considered a promising early biomarker of cognitive impairment in populations without dementia or stroke [42]. Furthermore, HRV was suggested as a marker of self-regulatory processes in neurodegenerative conditions [43]. The association between higher HRV and better cognitive performance was also confirmed in a multi‐ethnic cohort of aging adults [44] and across groups of different fitness levels [45,46]. In addition to heart rate zones, another top heart feature is variability of resting heart rate. A higher resting heart rate has been associated with an increased risk of cognitive decline [47-49].

The top physical activity features were light physical activity (distance and time). Several studies have established the relationship between light physical activity and risk for dementia. Increased physical activity level, including a low amount of light-intensity physical activity, has been associated with a reduced risk of dementia [50], and increasing light physical activity helps to maintain cognitive function among community-dwelling older adults [51]. Additionally, light activity as low as 10 minutes per day measured through accelerometers was previously shown to be associated with better cognitive function, while light physical activity performed ≥3 hours per day could be a protective factor for maintaining cognitive function in older adults [52,53].

Finally, the top feature associated with sleep was the average length of deep sleep. A comparison of Fitbit’s deep sleep stage with EEG signals found that it aligns with the N3 stage of the NREM sleep stage [54]. There is interest around the role of N3 sleep in dementia given its pivotal role in memory consolidation, but evidence about this association is still limited [55]. One study that focused only on patients with obstructive sleep apnea found that a shortened N3 stage of sleep led to declarative memory deficit [56].

Although all but 1 MCI participant had occasional naps, only one-half of the controls had naps. Focusing only on the participants who had naps, we obtained good performance, suggesting that information about naps could be important for identifying MCI status, where the length of the nap is one of the selected predictors. Previous reports on the associations between naps and cognitive function were inconclusive. No association was found between napping and cognitive decline in a meta-analysis of observational studies conducted with healthy individuals [57], and there was only a modest causal association between habitual daytime napping and larger total brain volume [58]. However, afternoon napping was related to better cognitive function in the aging Chinese population [59]. This has been related to higher levels of inflammatory markers that may induce cognitive impairment [60] and may disrupt circadian rhythms at the time of day of best coordination and fastest reaction time [61].

A major concern we had with this older adult population was whether they would continuously wear the fitness tracker and keep it charged. Fortunately, all participants except 1 who returned it early wore and charged their fitness tracker for the full term. When alerts were received that their battery was low, a research coordinator contacted the participant or their caregiver to remind them to recharge it.

### Limitations

Our study has 3 major limitations. The primary limitation of our pilot study is our small sample size and the limited racial diversity in our population. We attempted to address potential biases between the patients with MCI and controls by including couples, who were both age-matched but also had similar daily schedules. Although our selected features agree with established risk factors for cognitive impairment, the sample size may lead to 2 limitations, including the “curse of dimensionality,” where the number of features exceeds the number of samples, and co-linearity between different data types. For example, when a specific heart zone is elevated on a specific day, other zones will correspondingly be lower on the same day. We attempted to address the “curse of dimensionality” by having an independent step of feature selection that selected features that appear in more than 1 fold. A result of the sample size is limited generalizability. In order to generalize our MCI prediction models, larger and more diverse populations are needed to establish their use in the general population.

A second limitation is possible selection bias. Several potential participants declined to participate because they already had a fitness tracker or a smartwatch. It is possible that the population who already uses this technology are different in relevant physical activity traits than the population we recruited.

Finally, our clinical study may have interfering comorbidities and medications that may affect sleep; for example, 3 patients with MCI had sleep apnea and were taking medications that may affect sleep such as donepezil, rivastigmine, sertraline, escitalopram, melatonin, duloxetine, and gabapentin. Some of these medications, such as antidepressants (sertraline, escitalopram, and duloxetine) and gabapentin, have been reported to affect deep sleep, which is our prominent sleep feature [62-65], while the rest primarily influence only the REM stage [66-73].

### Conclusions

Our pilot study demonstrates the use of fitness trackers for determining MCI status. This study is intriguing in that it suggests that it may be possible to use an inexpensive, convenient digital biomarker applied serially to achieve the critical goal of detecting MCI due to AD pathology as early as possible because that is when new disease-modifying therapies have the greatest efficacy. Clearly, more study is necessary to ascertain the sensitivity and specificity of this digital biomarker. Another important goal of this study is to continue to follow these and other participants to learn more about ongoing digital biomarker measurements as they relate to future cognitive decline.

## Appendix

Multimedia Appendix 1 Supplementary Tables and Figures.

## Abbreviations

AD: Alzheimer disease
AUC: area under the curve
EEG: electroencephalogram
HRV: heart rate variability
MCI: mild cognitive impairment
MRI: magnetic resonance imaging
NDC: Neurosciences Neurocognitive Disorders Center
NREM: nonrapid eye movement
PET: positron emission tomography
REM: rapid eye movement
SHAP: Shapley Additive Explanations
SVM: Support Vector Machines
UTHealth: University of Texas Health Science Center at Houston

## Data Availability

Deidentified data and code are available at [74].
