# Supplementary material for: Prediction of Mild Cognitive Impairment Status: Pilot Study of Machine Learning Models Based on Longitudinal Data From Fitness Trackers
Source: JMIR Form Res. 2024 Jul 18;8:e55575. doi: 10.2196/55575 (PMC11294783; doi:10.2196/55575)
Supplement: Multimedia Appendix 1 [file formative_v8i1e55575_app1.pdf]

Supplementary Material

Prediction of Mild Cognitive Impairment Status: Machine Learning Models Based on Longitudinal Data From Fitness Trackers

Tables

Table S1. Co-morbid conditions for the MCI patients. Co-morbidities that are unique to only one patient are omitted.

Comorbidities	Number of patients
Hyperlipidemia	11
Depression	7
Hypertension	6
Anxiety	4
Sleep Apnea	3
Gastroesophageal reflux disease (GERD)	2
Allergies	2
Diabetes	2
Small vessel ischemic disease (SVID)	2

Table S2. Medications prescribed for the MCI patients with prescription time overlapping with the time of wearing the fitness tracker. Medications taken by only one patient are omitted.

Medication	Number of patients
Aspirin	8
Donepezil	7
Memantine	5
Atorvastatin	7
Vitamin C	6
Multivitamin	5
Vitamin E	4
Rivastigmine	3
Acetaminophen	4
Sertraline	3
Omeprazole	3
Metoprolol Succinate	3
Vitamin B12	3
Vitamin D3	3
Escitalopram	2
Rosuvastatin	2
Melatonin	2

Fish Oil	2
Lumigan	2
Levocetirizine	1
Hydrocortisone Cream	1
Ibuprofen	2
Folic Acid	2
Lisinopril	2
Duloxetine	1
Gabapentin	1

Table S3. Performance of different machine learning algorithms with a 5-fold cross validation, averaged over five runs with different splits.

Data	CatBoost	RF	XGBoost	SVM	LR	GNB
Activities	0.7619	**0.8015**	0.6865	0.7658	0.6746	0.7361
Activities + Heart rate	0.8214	0.8055	0.8035	**0.9206**	0.8412	0.8849
Main sleep	0.8055	0.7976	0.6547	0.7658	0.7896	**0.8333**
Naps	0.9166	0.8888	0.7361	0.4166	0.7222	**0.9861**
Activities + Main sleep	**0.8730**	0.8659	0.7301	0.8670	0.8611	0.8106
Activities + Naps	**0.9722**	0.9027	0.9444	0.8823	0.8611	0.6805
Activities + Heart rate + Main sleep	**1.0**	0.9880	0.9365	0.9484	0.9365	0.9722
Activities + Heart rate + Naps	0.9444	0.9166	0.9027	0.8611	**0.9615**	0.7778

Table S4. Performance of different machine learning algorithms with leave-one-out cross validation.

Data	CatBoost	RF	XGBoost	SVM	LR	GNB
Activities	0.5833	0.5938	0.4688	0.6667	0.6979	**0.7396**
Activities + Heart rate	0.8229	0.7812	0.7292	**0.9167**	0.7812	0.8012
Main sleep	**0.7708**	0.7448	0.5312	0.5990	0.7396	0.6875
Naps	0.8636	0.7614	0.6591	0.0909	0.6136	**0.8864**
Activities + Main sleep	**0.8438**	0.7917	0.6667	0.6458	0.7708	0.7917
Activities + Naps	**0.9318**	0.8864	0.8636	0.8409	0.750	0.6818

Activities + Heart rate + Main sleep	**1.0**	0.9375	0.9167	0.9271	0.9271	0.9167
Activities + Heart rate + Naps	0.8864	0.8636	0.9091	0.8409	**0.9545**	0.6705

Table S5. Performance of different machine learning algorithms with majority vote.

Data	CatBoost	RF	XGBoost	SVM	LR	GNB
Activities	**0.7166**	**0.7166**	**0.7166**	**0.7166**	0.6666	0.6333
Activities + Heart rate	0.6333	**0.6833**	0.6666	**0.6833**	0.6166	0.5166
Main sleep	0.6458	0.7803	0.6666	**0.8541**	0.7916	0.50
Naps	0.4375	0.4583	0.4375	**0.50**	0.4583	0.3125
Activities + Main sleep	0.7666	**0.7833**	0.6250	0.7666	0.6916	0.6041
Activities + Naps	**0.750**	0.6666	0.6666	**0.750**	0.5833	0.4167
Activities + Heart rate + Main sleep	**0.8333**	0.80	0.7750	**0.8333**	0.7333	0.5999
Activities + Heart rate + Naps	0.6666	0.5833	0.50	**0.750**	**0.750**	0.50

Table S6. Selected features from each aggregated data type.

Data	# of selected important features	Selected important features
Activities	15	MAX(lightly active distance),<br>MAX(lightly active minutes),<br>MAX(sedentary minutes),<br>MEAN(lightly active distance),<br>MEAN(lightly active minutes),<br>MEAN(calories out),<br>MIN(sedentary minutes),<br>STD(lightly active distance),<br>STD(lightly active minutes)<br>STD(sedentary minutes),<br>SUM(lightly active minutes),<br>SKEW(calories out),

		SKEW(moderately active),<br>SKEW(fairly active minutes),<br>SKEW(very active minutes)
Activities + Heart rate	10	MAX(lightly active minutes),<br>SKEW(moderately active),<br>MEAN(heart rate zone fat burn calories out),<br>MEAN(heart rate zone fat burn minutes),<br>MEAN(resting heart rate),<br>MIN(resting heart rate),<br>SKEW(heart rate zone fat burn minutes),<br>STD(heart rate zone peak max),<br>STD(heart rate zone cardio min),<br>MEAN(heart rate zone out of range minutes)
Main sleep	15	MAX(light sleep count),<br>MAX(wake count),<br>MEAN(deep sleep minutes),<br>MEAN(deep sleep minutes (avg)),<br>SKEW(deep sleep minutes),<br>SKEW(total sleep records),<br>MAX(deep sleep minutes),<br>MAX(light sleep minutes (avg)),<br>MEAN(light sleep minutes),<br>MEAN(REM count),<br>MEAN(REM minutes (avg)),<br>SKEW(REM minutes (avg)),<br>STD(efficiency),<br>SUM(REM minutes),<br>MEAN(minutes asleep)
Naps	6	MEAN(restless count),<br>MIN(efficiency),<br>SKEW(time in bed),<br>STD(awake minutes),<br>STD(efficiency),<br>STD(restless count)
Activities + Main sleep	15	MAX(light sleep minutes(avg)),<br>MAX(wake count),<br>MAX(lightly active minutes),<br>MEAN(REM count),<br>MEAN(deep sleep minutes),<br>MEAN(deep sleep minutes(avg)),<br>MEAN(lightly active minutes),<br>SKEW(deep sleep minutes),<br>SKEW(fairly active minutes),<br>SKEW(REM minutes(avg)),<br>SKEW(moderately active),<br>SUM(lightly active distance),

		SUM(lightly active minutes),<br>SUM(sedentary minutes),<br>MIN(lightly active distance)
Activities + Naps	11	SKEW(lightly active distance),<br>SKEW(activity tracker),<br>SKEW(lightly active minutes),<br>SKEW(marginal calories),<br>SKEW(steps),<br>SKEW(time in bed),<br>SKEW(activity: total minutes),<br>SKEW(very active minutes),<br>STD(sedentary minutes),<br>SKEW(awake count),<br>SKEW(activity calories)
Activities + Heart rate + Main sleep	15	MAX(lightly active minutes),<br>MEAN(deep sleep minutes),<br>SKEW(heart rate zone fat burn calories out),<br>STD(heart rate zone peak max),<br>SUM(heart rate zone fat burn calories out),<br>SUM(heart rate zone fat burn minutes),<br>SUM(lightly active minutes),<br>MEAN(resting heart rate),<br>MIN(resting heart rate),<br>STD(heart rate zone cardio min),<br>SUM(lightly active distance),<br>SUM(resting heart rate),<br>MEAN(heart rate zone fat burn minutes),<br>SKEW(heart rate zone cardio min),<br>SKEW(heart rate zone peak max)
Activities + Heart rate + Naps	13	MAX(activity calories),<br>SKEW(lightly active distance),<br>SKEW(activity tracker),<br>SKEW(heart rate zone cardio minutes),<br>SKEW(lightly active minutes),<br>SKEW(marginal calories),<br>SKEW(steps),<br>SKEW(time in bed),<br>SKEW(activity: total minutes),<br>SKEW(very active minutes),<br>STD(lightly active distance),<br>SKEW(activity calories),<br>SKEW(fairly active minutes)

Table S7. Selected features from each daily data type.

**Data**	**# of selected important features**	**Selected important features**
Activities	7	activity calories,<br>calories out,<br>lightly active minutes,<br>marginal calories,<br>steps,<br>very active minutes,<br>lightly active distance
Activities + Heart rate	14	fairly active minutes,<br>marginal calories,<br>resting heart rate,<br>steps,<br>heart rate zone out of range calories out,<br>heart rate zone out of range minutes,<br>heart rate zone cardio calories out,<br>heart rate zone cardio minutes,<br>activity calories,<br>calories out,<br>lightly active minutes,<br>lightly active distance,<br>heart rate zone fat burn calories out,<br>heart rate zone fat burn minutes
Main sleep	7	total sleep records,<br>total minutes asleep,<br>light sleep count,<br>light sleep minutes(avg),<br>REM minutes(avg),<br>wake count,<br>wake minutes(avg)
Naps	4	total minutes asleep,<br>total time in bed,<br>restless count,<br>awake minutes
Activities + Main sleep	12	activity calories,<br>calories out,<br>lightly active minutes,<br>lightly active distance,<br>steps,<br>moderately active,<br>very active minutes,<br>light sleep minutes(avg),<br>deep sleep minutes(avg),<br>total time in bed,<br>rem minutes(avg),<br>total sleep records

Activities + Naps	13	activity calories,<br>calories out,<br>fairly active minutes.<br>lightly active minutes,<br>lightly active distance,<br>sedentary minutes,<br>steps,<br>very active minutes,<br>activity: total minutes,<br>activity tracker,<br>moderately active,<br>total minutes asleep,<br>total sleep records,
Activities + Heart rate + Main sleep	13	calories out,<br>marginal calories,<br>lightly active minutes,<br>very active,<br>resting heart rate,<br>heart rate zone out of range calories out,<br>heart rate zone out of range minutes,<br>heart rate zone fat burn minutes,<br>heart rate zone fat burn calories out,<br>heart rate zone cardio minutes,<br>heart rate zone cardio calories out,<br>REM minutes(avg),<br>light sleep minutes(avg)
Activities + Heart rate + Naps	13	calories out,<br>lightly active minutes,<br>sedentary minutes,<br>steps,<br>very active minutes,<br>resting heart rate,<br>heart rate zone out of range calories out,<br>heart rate zone out of range min,<br>heart rate zone out of range minutes,<br>heart rate zone fat burn max,<br>heart rate zone fat burn calories out,<br>heart rate zone cardio minutes,<br>total time in bed

Supplementary Figures

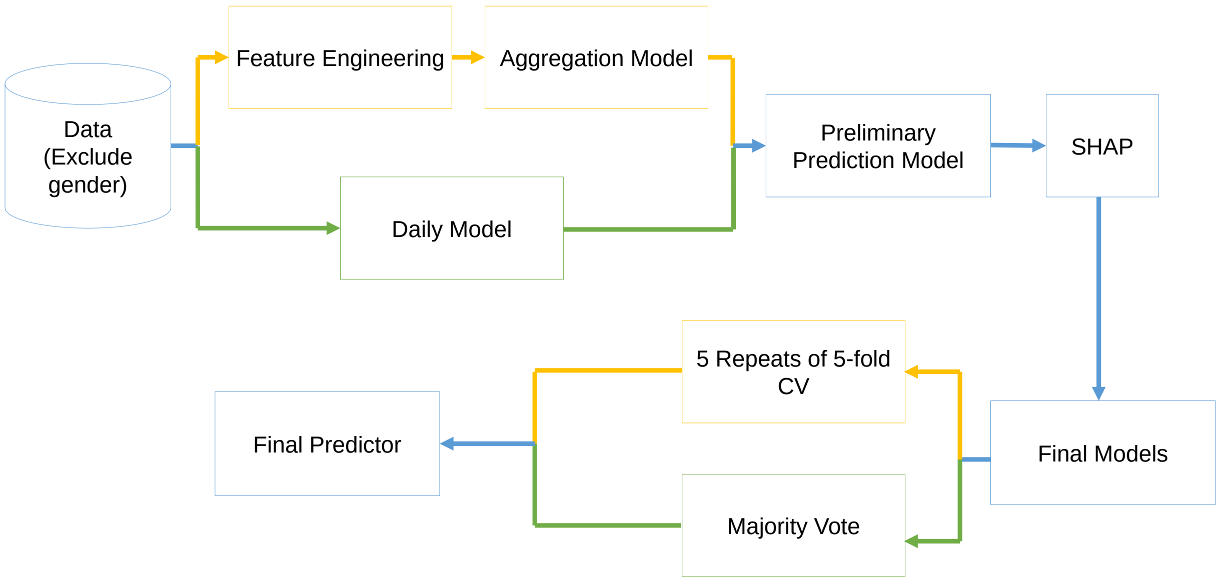

Figure S1. Overall processing steps

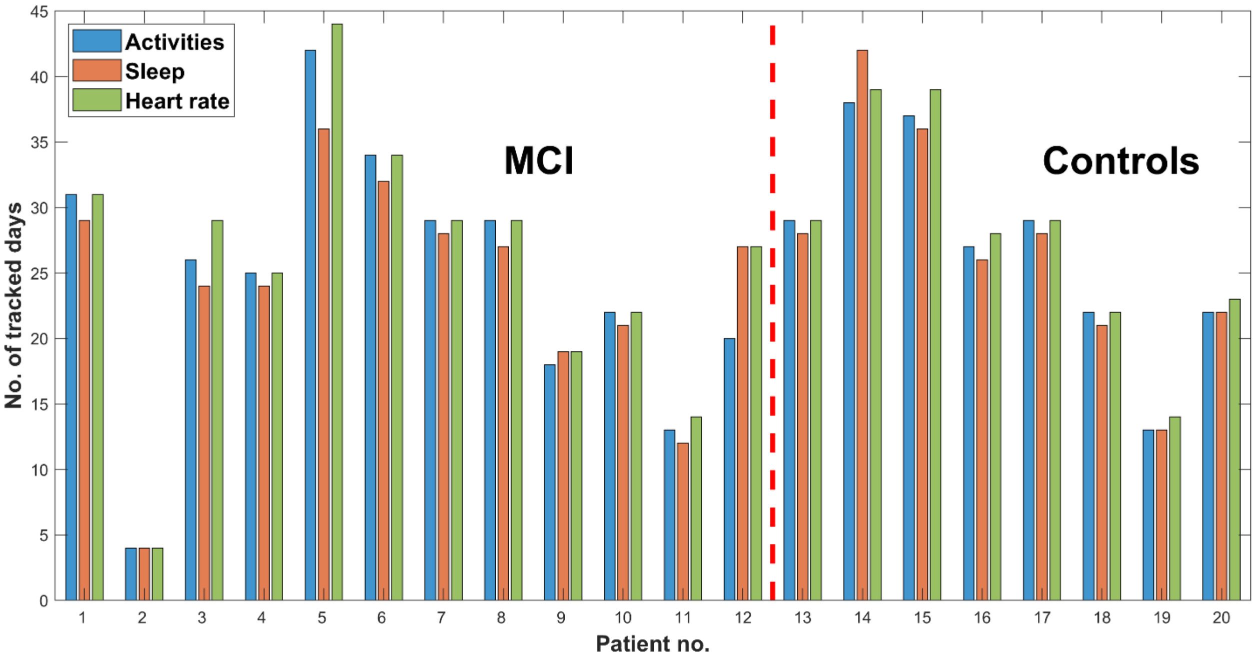

Figure S2. The number of recorded days per MCI (patients 1-12) and control patients (patients 13-20).

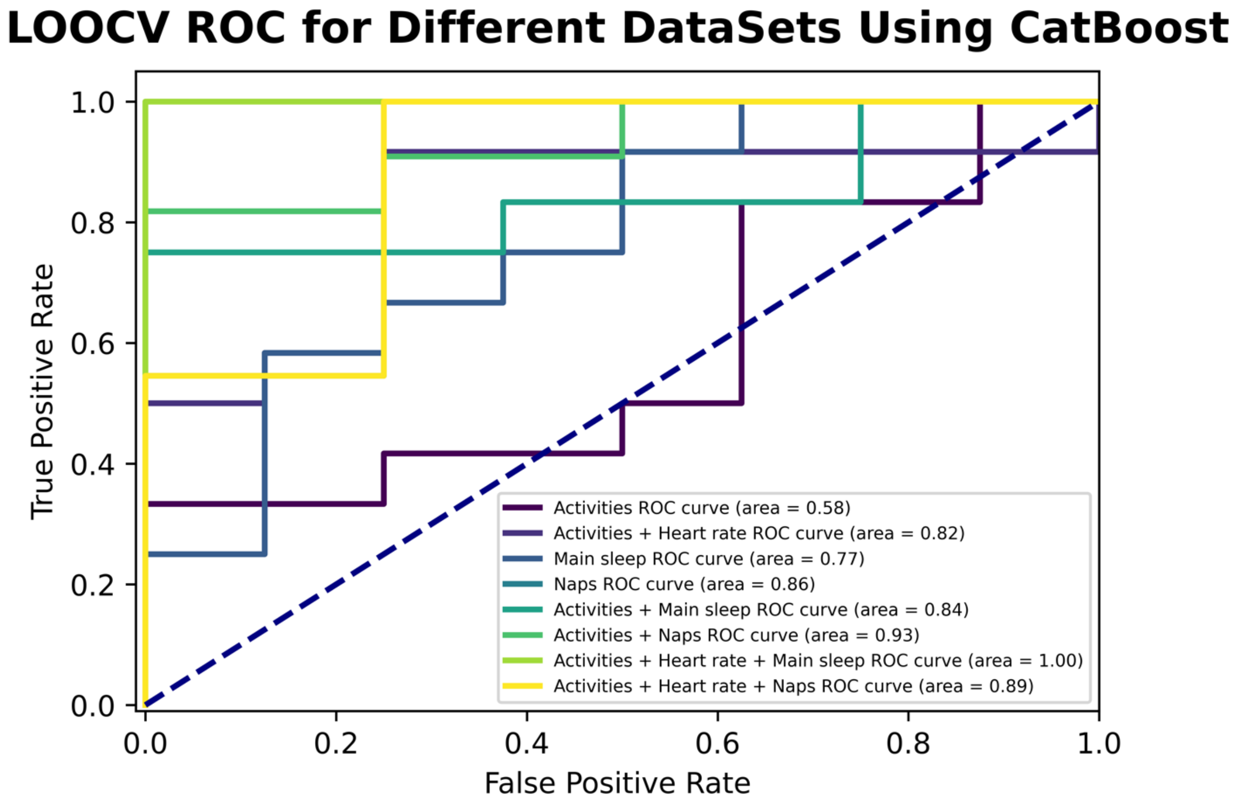

Fig. S3. Receiver operating characteristic curve of different modalities measured by fitness trackers for leave-one-out cross validation.
